# Multi-Criteria Decision Making Approaches Applied to Waste Electrical and Electronic Equipment (WEEE): A Comprehensive Literature Review

**DOI:** 10.3390/toxics9010013

**Published:** 2021-01-18

**Authors:** Samuele Marinello, Rita Gamberini

**Affiliations:** 1 En&Tech Interdepartmental Center, University of Modena and Reggio Emilia, Piazzale Europa 1, 42124 Reggio Emilia, Italy; rita.gamberini@unimore.it; 2 Department of Sciences and Methods for Engineering, University of Modena and Reggio Emilia, Via Amendola 2, Padiglione Morselli, 42122 Reggio Emilia, Italy

**Keywords:** e-waste management, waste electrical and electronic equipment (WEEE), decision support model, multi-criteria decision making, literature review

## Abstract

The global demand for electrical and electronic equipment has undergone continuous growth in recent years due to the effect of industrialization and technological development. This indicates substantial quantities of e-waste that need to be managed properly to reduce their environmental impact and to avoid inappropriate forms of disposal. The purpose of this paper is to review the most popular multi-criteria decision-making approaches applied to the management of waste electrical and electronic equipment, analyzing how they are used to contribute to the improvement of management strategies along the entire supply chain. For this purpose, a methodological protocol for the collection, selection, and analysis of the scientific literature was applied, identifying 44 papers on which to conduct this study. The results showed that numerous authors have developed multi-criteria approaches, with particular attention to recycling phase. The analytic hierarchy process is the most widespread multi-criteria approach, often coupled with VIKOR, DELPHI, and TOPSIS methods. The numerous decision making criteria adopted cover different reference dimensions: environmental, economic, social, technical, and legal. Considering environmental aspects also in decision making processes means enhancing the relevance of this dimension, as well as encouraging practices that reduce the impact of toxic substances on the environment and living organisms.

## 1. Introduction

Electrical and electronic equipment (EEE), as reported by the European Directive on Waste Electrical and Electronic Equipment (WEEE), is defined as “equipment that is dependent on electric currents or electromagnetic fields in order to work properly and equipment for the generation, transfer and measurement of such currents and fields and designed for use with a voltage rating not exceeding 1000 volts for alternating current and 1500 volts for direct current” [1]. They are products that are widely spread thanks to their extensive daily use, from work activities to daily life [2]. As reported by [3], over 900 different EEE items can be found in global markets. This scenario is closely linked to the growth of industrialization and the technological development that have affected the whole world, which have determined a growing demand for EEE with few repair options and which, in many cases, have become obsolete in a very short time (often much earlier with respect to their technical life) [4,5,6,7]. In [8], the authors underline how the life expectancy of (especially) small electronic devices is becoming shorter and shorter. The authors reported the estimated lifespan of different EEE, starting at 4.6 years for smartphones and reaching 7.4 years for flat TVs.

Once obsolete or non-functional, EEE must be discarded, becoming what is defined as WEEE [9]. WEEE or e-waste includes all types of e-products, and their parts, that have been discarded as waste [2]. They can be of different nature and types. 

WEEE are differently classified. For example, at the European level, the WEEE Directive 2012/19/EU-Annex III classifies them into six categories [1,10]:

1. Temperature exchange equipment;
2. Screens, monitors, and equipment containing screens;
3. Lamps;
4. Large equipment;
5. Small equipment;
6. Small IT and telecommunication equipment.

Classification does not follow an internationally uniform pattern. For example, in China, the subdivision of WEEE uses fourteen classes: televisions, refrigerators, washing machines, air conditioners, personal computers, range hoods, electric water heaters, gas water heaters, fax machines, mobile phones, single-machine telephones, printers, copiers, and monitors [11]. 

**Figure 1:**
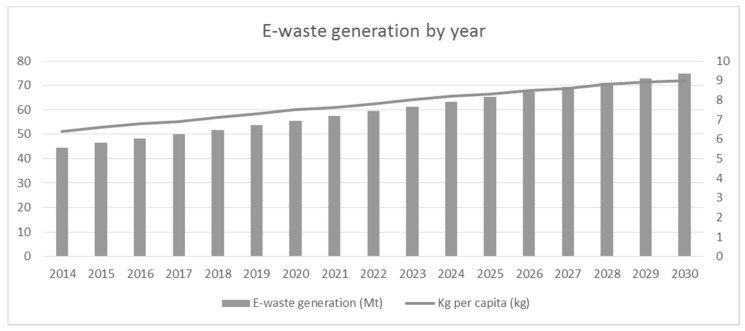
Trend of global Waste Electrical and Electronic Equipment production, overall and per capita (adaptation by the authors of the data from [11]).

Globally, their diffusion is taking on extremely significant numbers. Reporting the data described in the monitor report produced by [11], globally, the total EEE consumption is increasing annually by 2.5 million metric tons (Mt), reaching an overall e-waste production of about 53.6 Mt, with a per capita value of about 7.3 kg. This trend is continuously growing, with a projection of doubling these quantities by 2030 in just 16 years, as reported in Figure 1.

Asia, the Americas, and Europe are the three main areas that generate the largest quantities, with 24.9, 13.1, and 12 Mt, respectively. Oceania and Africa are less impactful, with 2.9 and 0.7 Mt, respectively. Analyzing the per capita data, the ranking of geographic areas changes, with Europe in first place (16.2 kg), followed by Oceania (16.1 kg), the Americas (13.3 kg), Asia (5.6 kg), and Africa (2.5 kg) [11].

The difficulties associated with the management of e-waste are manifold. First of all, it constitutes a type of waste that is extremely difficult to treat because it contains a heterogeneous mix of components and materials: ferrous metals, non-ferrous metals, glass, plastics, other materials [10]. Many of these contain hazardous substances that are toxic to the environment and to human health (in particular, mercury, brominated flame retardants (BFRs), chlorofluorocarbons (CFCs), and hydrochlorofluorocarbons (HCFCs)) and are not biodegradable over time [12,13,14]. The topic of hazardous substances is essential and is the basis for the REACH regulation (at the European level) and, from 2021, for SCIP database, which will improve transparency on hazardous substances in articles.

Some of the migration processes of pollutants from WEEE (or from management processes) towards environmental and biological receptors follow different paths, as summarized in [11,15,16,17]. Below is the list described by [11]:

- Dumping acid used to remove gold into rivers;
- Leaching of substances from landfills or stored electronics;
- Particulate matter, dioxins, furans from dismantling electronics;
- Contaminants entering the water system and food system through livestock, fish, and crops;
- Exposure through food, water, air;
- Home based workshops;
- Inhaling fumes from burning wires and cooking circuit boards;
- Pregnant women working as recyclers;
- Ingesting contaminated dust on surfaces;
- Playing with dismantled electronics;
- Children and adolescents working in collection, dismantling, and recycling.

At the international level, the approach to their management has significantly changed thanks to an increase in environmental awareness, enforced legislation, industrial ecology, and corporate citizenship [18,19,20]. Several risk assessment tools are available that apply, for example, matrix methods, fault tree analysis, and event tree analysis [21]. The Life Cycle Assessment (LCA) is a widely used tool to support risk assessment, considering several issues. In the research of [22], an overview of the LCA studies applied to the management of WEEE is reported, indicating the different sectors of intervention of each study. Many experiences have been applied in some countries, e.g., [23] applies the LCA approach to the WEEE sector in Belgium, [24] uses it in the UK, and [25] in Korea. As reported by [26], the Multi-Criteria Decision Making (MCDM) approaches also contribute to managing environmental risks through their ability to analyze various aspects at the same time (including environmental aspects).

At the regulatory level, countries are not all aligned globally. Europe has a highly developed regulatory system, thanks to the WEEE Directive [1], which regulates the collection and recycling of WEEE for all member states through reference quantitative targets. The Directive intends to favor the minimization of WEEE production, favoring sustainable approaches through greater reuse, recycling, and material recovery. Each member state is assigned the task of establishing national strategies and tools to achieve community goals through a shared effort. In India, which still suffers greatly from the informal sector, the E-waste Management and Handling Rules were adopted in 2012, already considering the EPR framework and setting objectives for the recovery and recycling of WEEE. In the USA, the legislation for the regulation of WEEE is entrusted to each individual state. Not all states have adopted legislation and there is a heterogeneous mosaic [27]. The National Solid Waste Policy (Waste Law) was adopted in Brazil, which encourages the development of reverse logistic initiatives for the recovery of WEEE. In Africa, only a few countries have WEEE regulations (e.g., Egypt, Ghana, Madagascar, Nigeria, Rwanda, South Africa, Cameroon, Côte D’Ivoire), but many still do not. These are some examples of the international regulatory environment. A broader treatment is described by [10,11].

Despite this, currently, the management of this waste, which includes the collection, transport, processing, recycling, and export or disposal of waste materials [28], represents a global challenge. Industrialized countries with established waste management systems often find trouble with the complex nature of WEEE, while in less developed countries, where waste management structures are scarce or non-existent, the difficulties are amplified and further aggravate the whole local waste management sector [29,30,31]. In these less industrialized countries, e-waste is managed mostly by the informal sector. As reported by [9], about 80% of WEEE is illegally exported from developed to developing nations thanks to inexpensive workmanship and a lack of regulation and norms. Furthermore, a low percentage of the e-waste collected and recycled is formally documented: about 17.4% for the year 2019, with Europe having the highest collection and recycling rate (42.5%), while Africa had the lowest rate (0.9%). The remaining 82.6% of global e-waste is managed in an uncertain and poorly documented manner.

All this represents a huge wasted potential, even in green and circular economy approaches. In fact, WEEE is a source of important and valuable resources which can be used as secondary materials [32,33]. As reported by [11], WEEE may contain over 69 elements from the periodic table. Some are precious metals (gold, silver, copper, platinum, palladium, ruthenium, rhodium, iridium, and osmium), others are critical raw materials (cobalt, palladium, indium, germanium, bismuth, and antimony), and others are noncritical metals (aluminum and iron). Globally, the economic value of the quantities of WEEE estimated for 2019 is about USD 57 billion, while the amount of raw materials is 25 Mt [11]. Many of these materials are recognized as critical raw materials and, therefore, alternative sources can significantly reduce the criticalities and strong pressures on traditional mines. To testify to this, the European Community has included several materials that could be recovered from WEEE within its list of 30 critical raw materials, as updated in 2020 [34]. Through the exploitation of WEEE as mines of secondary raw materials, countries can benefit from some important benefits, such as the recovery of precious materials, economic advantages, environmental benefits, and a reduction in dependence on countries supplying raw materials [11].

**Figure 2:**
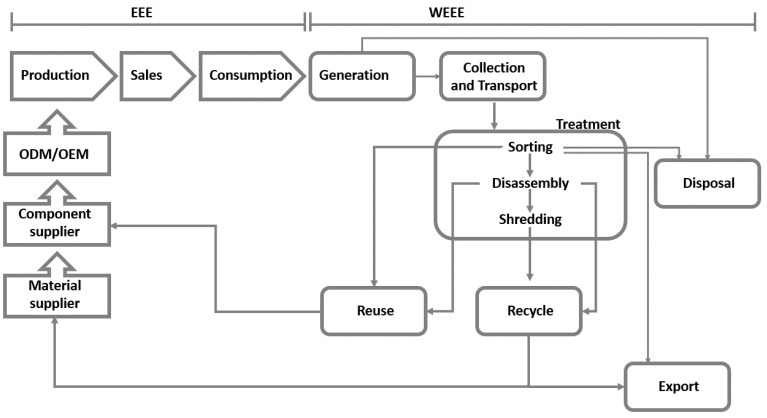
Supply chain structure.

As reported by [35,36], it is necessary to better disseminate waste management approaches, optimizing current practices or designing specific solutions according to the needs and characteristics of different territories. The need is to operate in the different phases that make up the EEE supply chain and the reverse logistic of WEEE (Figure 2). This is a primary challenge, especially in developing countries [37].

Since operating in the context of e-waste management requires very complex and very expensive infrastructures, technologies, and operational procedures [7], the planning, design, and optimization of the various phases of the supply chain require the support of management tools that help governments and stakeholders with related decision making aspects. Among these, life cycle assessment (LCA), material flow analysis (MFA), multi-criteria decision making (MCDM), and extended producer responsibility (EPR) are some of the tools that contribute to the solution of decisional problems in the management of e-waste problems [12]. Each tool contributes to the knowledge and deepens specific aspects that characterize the problem of WEEE management, focusing on characteristic aspects. The LCA approach concentrates on the analysis of impacts on specific environmental categories by analyzing flows and processes; MFA analyzes the flows of materials, defining their interaction with the different environmental matrices. LCA and MFA have several common aspects, as well as being able to interact easily with each other. EPR uses environmental impact tests to address legal issues. MCDM, thanks to the wide flexibility of the models, can interact and integrate with all the other tools, expanding the scope of the evaluations in different fields (e.g., economic, managerial, social, technical). This allows models to analyze a problem from different points of view at the same time.

The purpose of the paper is to review the most popular MCDM applied to the management of WEEE, analyzing how they are used to contribute to the improvement of management strategies along the entire supply chain. The intended purpose is to promote knowledge of the MCDM models that have been applied to the management of WEEE, highlighting which are the most widespread decision making criteria and which are the approaches adopted by the authors to combine them with each other in the search for an optimal solution. Particular attention has been paid to the environmental dimension, to emphasize how the use of MCDM can help to make the management of WEEE less dangerous for the environment quality and for the health of living organisms. The choice of WEEE as the subject of research is linked to their growing diffusion, to the potential negative impacts due to incorrect management, and to their great potential as mines of important materials in the context of circular economy and sustainable development strategies. The management of WEEE, given its heterogeneous nature and the complexity of its supply chain, has been the subject of numerous research studies that have applied various tools and methodological approaches for the study and optimization of management procedures. The objective of this review paper is to collect and critically describe the studies that have applied MCDM approaches to the study of e-waste and to the best configuration of specific aspects that make up their supply chain.

The review aims to answer the following research questions:

− What are the main aspects of the WEEE supply chain that are addressed with MCDM tools?
− What are the most widely used MCDM approaches?
− What could be the future lines of research and development of MCDM approaches applied to the WEEE sector?

The work is organized as follows: in Section 2, the applied methodology for the literature review is reported; Section 3 provides a description and a discussion of the results; and finally, Section 4 outlines some conclusions.

## 2. Materials and Methods 

**Figure 3:**
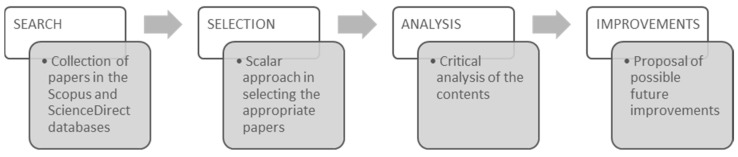
Methodological steps used to develop the proposed review work.

The work described has been structured according to a four-step approach (Figure 3), which, starting from the collection of works available in the literature, explored the aspects related to the use of MCDMs in the management of WEEE to identify possible research gaps and suggest possible future research directions.

Decision making theory uses explicit models to obtain answers to questions posed by a stakeholder, in order to make a decision making process more systematic and rational and to favor behavior that increases the coherence between the decision making process and the established objectives [38,39]. A multi-criteria problem can be defined as a decision making problem when characterized by the following aspects: the need to operate by choosing, ranking, sorting, or describing a finite or infinite number of potential or alternative actions; the existence of at least two decision criteria; the presence of at least one decision maker [40]. These techniques allow the process of choosing to be rationalized by optimizing a vector of multiple criteria, weighed according to the priorities declared. The most common approaches applied in the literature include:

- The Analytic Hierarchy Process (AHP) is able to describe a complex problem through a hierarchical structure of the relationships between objectives, criteria, sub-criteria, and alternatives. Through the AHP approach, a complex decision making or planning problem is divided into its components or levels, which are ordered in an ascending hierarchical order. Elements and levels are compared to each other and related to an adjacent upper level. The final result is a set of priorities of relative importance between the various actions or alternatives [41].
- ELimination Et Choix Traduisant la REalité (ELECTRE) assigns higher ranks to alternatives that are preferred in most criteria and pass acceptable levels on all criteria at the same time [42].
- Decision Making Trial and Evaluation Laboratory (DEMATEL) evaluates the causal interdependence and association among the problem’s variables that is quantified on a scale of 0 to 4 [9].
- PROMETHEE is an outranking method that ranks alternatives on their deviation from the optimal point according to each criterion [43].
- The DELPHI method repeatedly collects expert opinions until a widespread consensus is reached with respect to the object of choice [44].
- Technique for Order of Preference by Similarity to Ideal Solution (TOPSIS) is a method that allows possible alternatives to be prioritized with the shortest distance from the ideal option and the greatest distance from the most disadvantageous, using appropriately quantified weights.
- Vlse Kriterijumska Optimizacija Kompromisno Resenje (VIKOR) is an MCDM approach for optimizing multi-criteria problems of a complex system by selecting the alternative deemed most efficient from a set of different possibilities. Choices are made through a ranking index on the basis of closeness to the ideal solution.

### 2.1. Material Search 

The methodological approach adopted in this review work to search research papers is shown in Table 1. Single journal articles, conference papers, and book chapters were the basic units of the research. The databases used to search for the material were Scopus and ScienceDirect. No restrictions were imposed on the journal, year of publication, or type of article. The only constraint set was to view articles written in English. The search for the papers was completed on 25 November 2020.

The keywords applied for the search were divided into two groups, referred to as “Group A” and “Group B”. The first group focused research on the primary object of the study (WEEE), while the second group specialized in the MCDM tools. Each keyword in Group A was searched coupled with the others in Group B using the “*” wildcard character employed for multi-word searches.

### 2.2. Material Selection

Despite the wide availability of papers, many have been excluded from the review because they are not relevant to the topic addressed in this research. This screening has been possible through the application of a scalar selection approach consisting of several steps, as reported in Table 2. An increasingly in-depth evaluation of the contents that characterize the paper allowed the authors to evaluate in greater detail whether it can be useful to respond to the research questions identified.

The first evaluation is conducted on the authors, on the date of the paper’s publication, and on the title of the research, which allow any duplicates to be identified and therefore eliminated. For the papers remaining after the first selection phase, the analysis of keywords and abstracts is completed. This evaluation allows a first analysis of the contents of the paper, with a view to understanding its structure, the approaches applied, and the main results achieved. For the remaining papers, some inclusion criteria were applied to verify whether the topics analyzed were consistent with the research. Specifically, the criteria applied were intended to select only the papers that deal exclusively with WEEE through the MCDM approach.

Only the articles that passed all these stages were subjected to a complete analysis of the entire text. To further enrich the selected papers, a final research and selection activity was applied through two approaches: browsing other known references and tracking down references in the selected papers (backward snowballing).

The papers identified through these types of selection were also subjected to all the steps of the selection phase. Overall, 44 papers were selected to be the subject of this review.

### 2.3. Material Analysis

The identified papers were the subject of the entire analysis work, through a descriptive and analytical approach, which is outlined in Table 3.

The descriptive analysis is aimed at providing an overall evaluation of the most representative elements of the selected reference literature, reporting, for each author, the title, the journal, the date of publication, the country of origin of the corresponding author, the type of publication and its origin (expressed as a form of research).

The analytical analysis, on the other hand, is aimed at exploring the contents of each article in greater detail, analyzing the phase of the supply chain in which the MCDM approach is applied, the type of MCDM approach used, the type of decision making criteria applied, and finally, the case study application.

## 3. Results and Discussion

### 3.1. Descriptive Analysis

**Figure 4:**
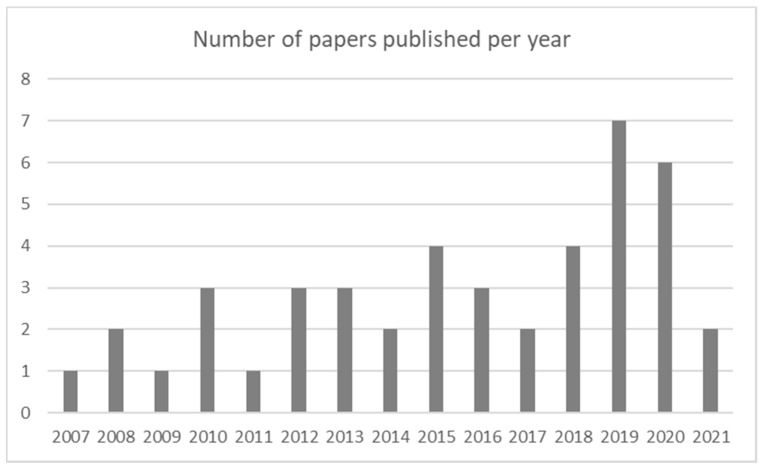
Number of papers published per year.

The selected articles describe research activities that were published during the period 2007–2021. Papers were collected for each year with at least one article, up to a maximum of seven articles per year. The years 2019 and 2020 are richer in terms of contributions than previous periods. Figure 4 reports their temporal distribution.

**Figure 5:**
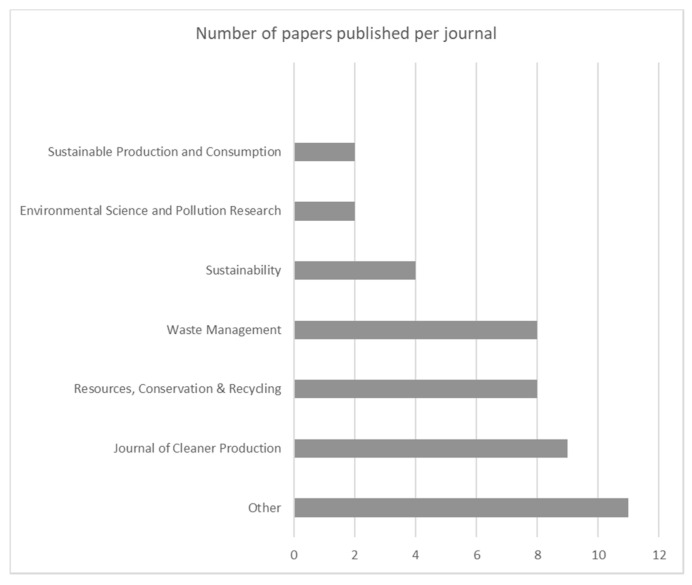
Number of papers published per journal.

Figure 5 reports the publication journal for each paper. From the distribution reported, the authors’ preference for three journals is evident: “Journal of Cleaner Production”, “Waste Management”, and “Resources, Conservation & Recycling”, with 9, 8, and 8 papers published, respectively. Almost 57% of the selected papers are concentrated in these three journals. The “Other” class includes all journals that have only one publication each. Altogether, this category includes 11 different journals.

**Figure 6:**
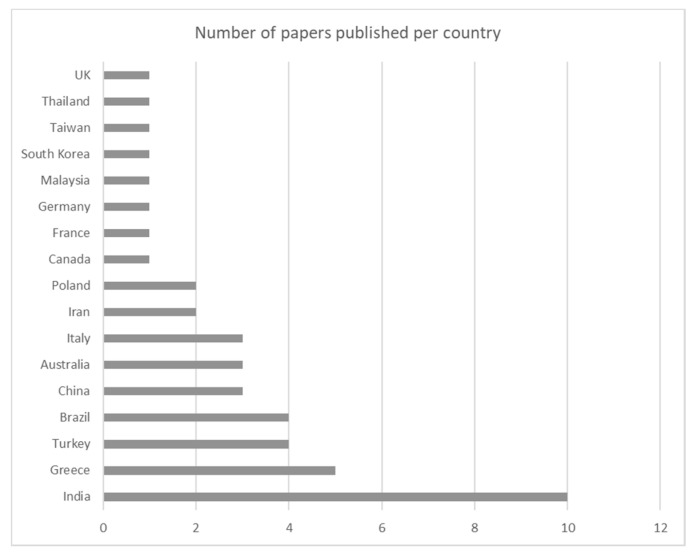
Number of papers published per country.

Figure 6 shows the distribution of the papers with respect to the author’s country of origin. India is the country of origin of most of the authors (about 24%), followed by Greece (12%) and Turkey (10%). More detached, with three papers each, are Italy, China, Australia, and Brazil. Then, there are 10 other countries with one or two contributions each.

Comparing the 44 selected articles, all of them can be classified as “research papers”; 32 come from the search and selection protocol, two from the browse approach, and the other 11 from the snowball method, as reported in Table 4.

### 3.2. Analytical Analysis

The first aspect investigated through analytical analysis is the WEEE management process analyzed through the use of MCDM approaches. The possible processes considered are the following: collection, transportation/storage, treatment (recycling, reuse, and disposal through incineration or landfill), and finally, export. Table 5 shows the supply chain phases analyzed through MCDM approaches. First, there are the papers that deal with the entire supply chain, followed by those that deal only with one or some phase(s). The lines have a width equal to the sections of the supply chain covered: Ref. [45] analyzes the entire supply chain, while [7] analyzes only the collection and transportation phase.

From the results reported in Table 5, it is clear that the specific phase of WEEE treatment with the purpose of recycling constitutes the activity most investigated by the authors analyzed: about 88% of the papers analyze this aspect. The other processes have similar percentages: collection has a frequency of 60%, transportation and storage about 69%, reuse about 69%, disposal about 64%, and export about 52%. It is important to underline that numerous authors simultaneously analyze different phases of the management process. In particular, 20 papers deal with the entire supply chain (from collection to export) and constitute almost half of the authors analyzed (48%), while 7% (3 papers) simultaneously analyze the collection and transport/storage phases. With the aim of increasing the WEEE collection rate, the authors in [62] have developed a model to define the on-demand WEEE take-back scheme from household residents through modern communication channels such as websites or mobile phone applications. The mathematical representation of the developed model was experimentally applied in the suburb of Tychy, a city in the Silesian region of Poland. This allows the management costs of the WEEE collection and management process to be optimized, ensuring an adequate number of vehicles and employees, and minimizing the length of routes, fuel consumption, and pollutant emissions.

Similarly, the authors in [63] developed a multi-criteria approach to optimize costs for picking up on demand WEEE from residents or stores. This model optimizes vehicle routes, the number of vehicles and the loading problem, ensuring the timeliness of the service. This approach was applied in the city of Opole (Poland). The authors in [7], analyzing the costs of the reverse logistics in the management of WEEE, orient the evaluations to the type of carrier that must be used with the use of different types of containers. The applied multi-criteria optimization model, in the choice between multi-type carriers of WEEE, optimizes the total logistical costs, the fuel consumption and the pollutant emissions. Infrastructure at collection points and recycling facilities is also considered in the selection process.

Five per cent of the papers (two authors) analyze the transport phase. In the study presented by [64], the authors describe how to manage the transport phase of hazardous WEEE through suitable carriers to reduce the potential risk to human health and the environment. Through MCDM approaches, the authors evaluated how to optimize the choice of hazardous WEEE carriers, describing a practical application in Turkey. The authors in [65] analyze the problem of the optimization of a WEEE transportation network by analyzing technical and environmental performance criteria. Starting from several municipal WEEE collection points and from one treatment plant, the study optimizes the use of collection vehicles and their paths.

The entire (and only) treatment phase (considering the recycling, reuse, and disposal phases) is analyzed by 7% of the authors. In particular, the authors in [70] apply the ELECTRE III approach to define the optimal location of WEEE treatment plants. Given the high costs involved in building the infrastructure and the need to ensure the efficiency and effectiveness of the WEEE treatment process, the optimal location requires accurate assessments. The authors in [28], using a fuzzy linear programming technique for the multidimensional analysis of preference (LINMAP) method, evaluate different alternatives for the management of WEEE, assessing their effectiveness. The best alternative involves the reuse and recycling of WEEE delivered to the treatment plants. The authors in [76] analyze the specific aspect of copper management of WEEE at the national level in France, evaluating in particular the costs of its management and the related environmental impacts.

Several authors (about 17%) focus their studies on the recycling phase only (as a subphase of the treatment). The authors in [14] analyze the metal recovery opportunities from WEEE by evaluating the characteristics of the products entering the treatment process. The authors in [73] deal specifically with the aspect of an extended polluter responsibility policy, supporting manufacturers in their decision making for optimal end-of-life alternatives. The authors in [5] identify additional potential candidate products that are outside of the current Australian National Television and Computer Recycling Scheme through an AHP-rating model. The authors in [72], studying the management of the WEEE recycling phase, support the definition of facility location and the national WEEE management policy. The authors in [74] apply the analytic hierarchy process (AHP) to evaluate the addition of new mandatory recycled waste to the WEEE list. The authors in [69] apply the multi-criteria group decision making approach to evaluate WEEE recycling programs, ensuring adequate consideration of all the interests of individual decision makers. The developed approach allows the concept of corporate sustainability to be incorporated into their regular planning decisions and business practices. The authors in [68] analyze a particular aspect of WEEE management and recycling: the use of robotic disassembly. In particular, the authors analyze the environmental benefits, technological feasibility, and economic viability of the application of this technological solution.

Through analytical analysis, the MCDM approach adopted by the selected authors was then analyzed as a second element of evaluation. The results are summarized in Table 6. First, there are the most analyzed MCDMs in the literature.

The MCDM approaches described by the selected papers are very rich and heterogeneous. Altogether, 18 different methodologies have been used. Within this scenario, some distinctive elements can be identified, as described below. The AHP [78] is the most widespread approach in the works analyzed in this review. This method is used by 32% of the selected authors. TOPSIS and VIKOR are both used by 12% of the authors. PROMETHEE is employed by 9% of the articles. The ELECTRE, DELPHI, and DEMATEL methods are less widely used. About 5% of the authors employ these types of approaches.

Numerous applications of multiple objective linear programming and fuzzy optimization methods are reported in the literature (17% is the frequency found). Finally, numerous other approaches have been described within the selected papers, but they have a low frequency, resulting in the use of only one author each. MAGIQ, MOGA, WSM, MAUT, ISC, and CPP are some of the approaches described.

Often, some of the approaches described have been applied in a combined way. The authors in [67] use the AHP, the analytic network process (ANP), ELECTRE, and PROMETHEE in order to determine the appropriate ranking of alternatives in the definition of the best WEEE management network through the use of 850 collection points for district municipalities and 15 large collection centers. ANP is an extension of AHP. The combined use of AHP and VIKOR was applied by 10% of the authors analyzed, with [46] analyzing possible solutions through solving the problems caused by the informal WEEE recycling on the environment and human health. The authors in [64] apply them under an intuitionistic fuzzy environment for the hazardous waste transportation selection process. The authors in [51] assess recycling partners’ alternatives with respect to green competency criteria. The authors in [52] apply these approaches for the selection of reverse logistics partners. The authors in [5] and [44] apply a combination of the AHP and DELPHI approaches. The former use them to select additional products to add to the WEEE list that makes up the Australian National Television and Computer Recycling Scheme. The second study uses them to determine the WEEE priority to be included in the extended producer responsibility system. Finally, the use of AHP and TOPSIS is described in the literature, in [47] for outsourcing choices, defining the reverse logistics service provider, while [18] applies them to overcome some of the barriers that make reverse logistics implementation difficult and hence reduce the success rate.

The third aspect studied with analytical analysis is the criteria used in the application of the multi-criteria approach. In particular, the results reported in Table 7 indicate the number of criteria applied by each author and their classification in dimensions that characterize environmental, economic, social, technical, and legal issues. The order in the table follows the number of criteria used in the papers.

Numerically, it can be observed that a very different number of criteria are used among the authors analyzed, ranging from 2 to 28 criteria. Ref. [55] applies the total distance travelled and greenhouse gas (GHG) emissions as criteria for determining the location of recycling centers. On the other hand, in [59,78], the authors employ a very large number of criteria for selecting a sustainable location for a WEEE recycling plant and for evaluating innovation strategies with respect to interacting sustainability-related criteria, respectively. About 80% of the papers analyzed use a number less than or equal to 10 decision criteria. Only 20% use more than 10 criteria and, of these, only 7% use more than 25. It is important to underline that some authors associate numerous other sub-criteria with each decision criterion. Ref. [19] is an example: it uses eight criteria, but considers 29 sub-criteria overall.

Compared to the five dimensions considered, only four papers apply criteria representative of all dimensions ([36,61,72,78]).

The most investigated domain is the technical one, with 86% of authors using criteria for this dimension. The economic dimension is in second place (70%), followed by the environmental one (61%), the social one (43%), and finally, the legal one (36%). The criteria used are shown in Table S1 of additional material.

The last aspect described with the analytical analysis is the description of the case study where the MCDM approach has been applied experimentally. The results are reported in Table S2 in the additional material (the order in the table follows the alphabetical order of the authors). Most of the authors (about 86%) applied the multi-criteria approach developed for territorial case studies. The other six authors (14%) described specific case studies without indicating territorial contexts. A significant example is the one described by [68], which refers to the evaluation of a laboratory-based robotic disassembly cell and three automotive electronic components.

**Figure 7:**
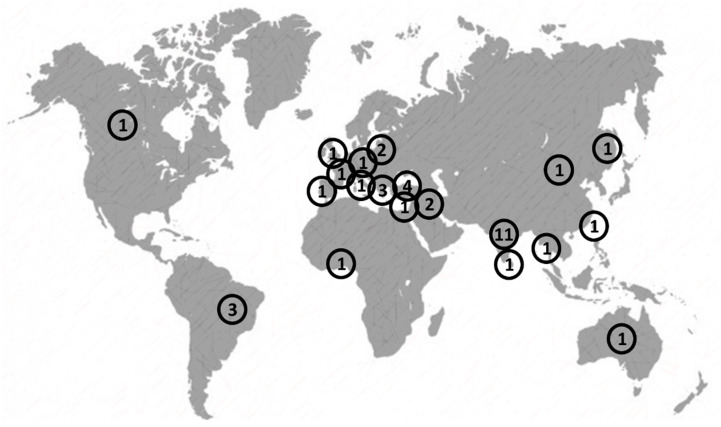
Spatial distribution of the case studies.

Analyzing their spatial distribution, Figure 7 reports the countries where multi-criteria approaches have been tested. India is the country with the most applications with nine papers, followed by Turkey with four studies, and Greece with three. The other countries have one or two studies each. The largest number of studies is in the European area.

## 4. Evaluation of Environmental Decision Criteria

As described in this paper, and as reported in Table 7 and in Table S1 in the additional material, several environmental aspects have been included as decision criteria in the MDCM approaches. The ability of a model to evaluate and integrate the environmental dimension among the decision making variables is an important aspect since:

- It recognizes the environmental issue as a significant decision making aspect, as well as economic, social, technical, and legal ones;
- It allows identification of an answer to the problem of WEEE management, improving its environmental impact, and reducing the effect that toxic substances can have on natural and biological receptors.

In particular, the variables of the described literature allow various factors to be analyzed. They guarantee an adequate distance from water bodies in order to minimize the major concern of pollution and toxicities of water resource [72]. Criteria for preserving water quality also allow for minimization of wastewater generation [47,75,77] and improvement in water purification and recycling [6]. Likewise, the impact of activities on air quality, soil and climate, as well as noise and odor emissions are considered in the decision process in order to reduce the impact on the environment and on the exposed population, as well as ensuring compliance with environmental regulations [54,77]. Similarly, solutions to collect and manage the hazardous substances and waste materials are underlined [47,68]. The ability to conserve resources (for example, through the percentage ratio of the returned products quantity over total EEE produced) is also a widespread factor [28] and allows the application of solutions that can encourage the recovery of resources as secondary raw materials. Similarly, minimizing the use of energy allows the achievement of economic and environmental objectives at the same time [75]. Preferring sites in areas far from biodiversity and natural habitats, reducing the possible interaction between WEEE and living organisms, helps to mitigate the possible toxic impact of substances released during e-waste management processes [72]. The widespread diffusion of criteria that refer directly to mandatory regulatory aspects, but also to voluntary green certification protocols, guarantee continuous improvement in performance and a competitive advantage on the reference markets [49,52,54,60,64]. The management of worker safety and health risk is another aspect that finds application of the MCDM, resulting very important in countries where legislation and controls are less developed [64].

## 5. Possible Future Developments

Some of the possible future developments that could favor the transfer and capitalization of the proposed approaches, as reported by the analyzed authors, are described below. The first concerns the exportability of the developed approach to other territorial contexts, keeping the structure of the approach unchanged, and making appropriate changes and adjustments to the special requirements of the problem in question: specific and strategic objectives, law issues, as well as the geographical characteristics of the area under consideration. The second possible development concerns the further implementation of the developed models, considering at the same time different aspects of WEEE management. Furthermore, the eco-design conception for EEE can be an aspect to be modelled to improve its reusability and recyclability. In the case of evaluations conducted with experimental data collected and processed at the “laboratory scale”, it would be important to test the methodologies developed through real cases. A further field of development concerns the integration of new criteria into the sets already used, covering the different dimensions identified in this study. The fourth aspect concerns the combined analysis of different MCDM approaches and the comparison of results with the sensitivity analysis and testing their performance in such a decision problem. Finally, the better ability to integrate quantitative data with the partial judgment of experienced stakeholders constitutes a further aspect that can be improved with future studies. Additionally, the vagueness/impreciseness of expert opinions in the evaluation process is an aspect that must be considered in the development and application of MCDM approaches.

## 6. Conclusions and Future Developments

This review has been implemented with the aim of collecting and describing, in a concise and organized way, the application of MCDM approaches to support the planning, design, and optimization of WEEE management. This paper has analyzed the management solutions through a general review of 44 selected papers.

Although EEE constitutes a particularly critical class of waste due to its impacts on the environment and health and, at the same time, is a very valuable source of secondary raw materials, its management is still very complex and often poorly documented, with much illicit trafficking from more developed countries to less rich ones. These aspects make the solution of the WEEE management problem still a long way off.

The analysis conducted in this paper made it possible to define how MCDM approaches can contribute to the management of WEEE through the integration of numerous criteria in decision making processes. In particular, the criteria applied by the authors reflect the importance that technical, economic, environmental, social, and legal aspects have in the management of WEEE, along the entire supply chain, with the recycling phase where significantly, the largest number of applications is concentrated.

India is the country of origin of most of the authors analyzed, followed by Greece and Turkey. This also reflects the spatial distribution of the case studies to which the developed MCDM approaches have been applied. It is important to underline that these areas have a high production of e-waste, but recycling rates and circular economy approaches are rather poor. Probably, this relevant problem (also considering the high rates of informal management, especially in India) has prompted researchers to analyze and look for better solutions.

## Figures and Tables

**Table 1:** Search phase approach.

**A**	**Research Questions**				
	- What are the main aspects of the WEEE supply chain that are addressed with MCDM tools? - What are the most widely used MCDM approaches? - What could be the future lines of research and development of MCDMs applied to the WEEE sector?				
**B**	**Database**				
	ScienceDirect				
	Scopus				
**C**	**Search Criteria**				
	Search unit	Single journal article/conference paper/book chapter			
	Journal	All			
	Year	All			
	Article type	All			
	Language	English			
	Date of search	25 November 2020			
**D**	**Keywords for Papers Identification**				
**Group A**		**Group B**	**ScienceDirect**	**Scopus**	**Total**
WEEE	AND	Multi-Criteria analysis	607	0	607
		Multi-Criteria Decision Making	429	0	429
		Decision support model	1198	23	1221
		Management	2775	945	3720
e-waste		Multi-Criteria analysis	59,138	19	59,157
		Multi-Criteria Decision Making	26,031	1	26,032
		Decision support model	77,062	19	77,081
		Management	210,204	1501	211,705
Waste Electrical and Electronic Equipment		Multi-Criteria analysis	5217	0	5217
		Multi-Criteria Decision Making	3025	0	3025
		Decision support model	6794	20	6814
		Management	13,730	1046	14,776
		**Total**	406,210	3574	409,784

**Table 2:** Selection phase approach.

**E**	**Steps for Material Selection**		
	Duplicate removal		
	Keywords and abstract assessment		
	Application of inclusion criteria		
		They only analyze WEEE	AND
		They apply MCDM exclusively	AND
		They present case studies	
	Full text assessment		
**F**	**Other Paper Sources**		
	From informal approach		
	From snowball method		

**Table 3:** Analysis protocol.

**I**	**Descriptive Analysis**	
	Year	
	Journal	
	Country	
	Type	Research paper
		Review
		Others
	Material collection	PD—Protocol-driven
		IA—Informal approaches
		SB—Snowball methods
**II**	**Analytical Analysis**	
	WEEE management process analyzed	
	MCDM approach	
	Type of decision criteria	
	Case study	

**Table 4:** Other paper sources used.

From search and selection protocol	31
From browse approach	2
From snowball methods	11
Total	44

**Table 5:** Supply chain phase analyzed through MCDM approaches.

Ref.	Collection	Transportation/Storage	Treatment			Export
			Recycling	Reuse	Disposal (Incineration, Landfill)	
[45]	Closed-Loop Supply Chain (CLSC)					
[46]	Barriers to the sustainable development of WEEE treatment industry					
[37]	WEEE reverse logistics model					
[47]	Evaluation and selection of third-party logistics service					
[48]	Selection of outsourcing firm for WEEE management					
[49]	Evaluation of alternatives for WEEE management					
[44]	Determine the WEEE priority to be included in the extended producer responsibility system					
[50]	Analysis of barriers affecting the implementation of WEEE management					
[9]	Analysis of barriers affecting the implementation of WEEE management					
[51]	WEEE recycling partner evaluation					
[18]	Prioritizing the solutions of reverse logistics					
[52]	Evaluation and selection of third-party reverse logistics partner					
[53]	Designing a sustainable recovery network					
[54]	A closed-loop supply chain with a circular economy approach					
[19]	Prioritizing solutions for reverse logistics barriers					
[55]	Clustering and reducing supply chains complexity					
[56]	Outsourcing contracts selection					
[6]	New scenarios assessment					
[57]	Prioritizing reverse logistics barriers					
[58]	Innovation strategies for reverse logistics					
[59]	Sustainable planning of WEEE recycling activities					
[60]	Interdependence among the e-waste mitigation strategies (MS) by cause/effect analysis					
[61]	Solutions for reverse logistics					
[7]	Type of carrier to be used					
[62]	Mobile collection with application of artificial intelligence					
[63]	WEEE collection on demand					
[36]	Improve WEEE management					
[64]	Select hazardous waste carriers					
[65]	Transportation network					
[66]	Evaluation of sites for the location of WEEE recycling plants					
[67]	Plant site selection					
[14]	Material recovery from WEEE					
[68]	Robotic disassembly to support recycling and recovery					
[69]	Evaluation the performance of WEEE recycling programs					
[70]	Units of Treatment and Recycling (UTR)					
[71]	Recover primary constituents from computers					
[72]	Location for Waste Electrical and Electronic Equipment (WEEE) recycling plant					
[28]	Assessment of three types of waste treatment					
[73]	Assess the residual value, environmental burden, weight, quantity, and ease of disassembly of each component					
[5]	Identify potential candidate products					
[74]	Identify potential candidate products					
[75]	Optimal WEEE management scheme among alternative options: recycling; reuse; disposal; export					
[76]	Best copper waste management model					
[77]	Alternative systems for the WEEE management					

**Table 6:** MCDM approach applied.

**Single Approach**	**Ref.**
AHP	[14,36,49,74]
Fuzzy optimization method	[45,63,65,69]
Multiple objective linear programming	[7,28,46,75]
DEMATEL	[9,51,55,61]
TOPSIS	[19,60,76]
PROMETHEE	[66,77]
CPP	[37,58]
ELECTRE III	[70]
WSM	[55]
RRR	[68]
MMM	[73]
ISC	[6]
MOGA	[53]
MAGIQ	[71]
**Combined Approach**	**Ref.**
AHP and VIKOR	[47,52,53,64]
AHP and TOPSIS	[18,48]
AHP and Delphi	[5,44]
BWM and VIKOR	[72]
AHP-Fuzzy	[49]
BOCR-TBL and ANP	[58]
AHP and ANP with ELECTRE and PROMETHEE	[67]
MAUT and PROMETHEE	[56]
Genetic Algorithms (GA) and GRASP	[61]

**Table 7:** Number and type of criteria used in the decision making phase.

Ref.	Number of Criteria	Criteria Type				
		Environmental	Economic	Social	Technical	Legal
[72]	28	X	X	X	X	X
[58]	28	X	X	X		
[48]	25	X	X	X	X	
[77]	17	X	X		X	X
[60]	15	X	X	X	X	X
[45]	14	X	X		X	
[64]	14	X	X	X	X	
[36]	12	X	X	X	X	X
[75]	12	X	X	X	X	
[37]	11		X	X		
[50]	10	X		X	X	X
[48,68]	10	X	X		X	
[66]	10		X		X	X
[54]	10	X			X	
[6]	9	X	X	X		
[71]	9				X	
[19,58]	8		X		X	X
[54,60]	8	X	X	X		
[28]	8	X	X		X	
[67]	7		X		X	X
[52]	7				X	
[9]	6		X	X	X	X
[49]	6	X	X	X	X	
[51]	6	X		X	X	X
[18]	6		X		X	X
[74]	6		X	X	X	
[14]	6				X	X
[46]	5	X	X	X	X	
[73]	5	X			X	
[76]	5		X			
[62]	5				X	
[69]	4	X	X	X	X	
[5,44]	4	X			X	
[70]	3		X		X	X
[7]	3	X	X			
[56]	3		X		X	
[61]	3		X		X	
[63,65]	3				X	
[55]	2	X			X	

## Data Availability

The data presented in this study are available in the paper and in the Supplementary Materials.

## Supplementary Materials

The following are available online at https://www.mdpi.com/2305-6304/9/1/13/s1, Table S1: Criteria used in the decision phase, Table S2: Case study description.
