# Supplementary material for: Multi-Criteria Decision Making Approaches Applied to Waste Electrical and Electronic Equipment (WEEE): A Comprehensive Literature Review
Source: Toxics. 2021 Jan 18;9(1):13. doi: 10.3390/toxics9010013 (PMC7831089; doi:10.3390/toxics9010013)
Supplement: Supplementary file 1 [file toxics-09-00013-s001.pdf]

Supplementary Materials: Multi-Criteria Decision Making Approaches Applied to Waste Electrical and Electronic Equipment (WEEE): A Comprehensive Literature Review

Samuele Marinello and Rita Gamberini

Table S1. Criteria used in the decision phase.

Environmental	Economic
• Consumption of fossil fuel<br>• Emissions due to transportation<br>• Energy consumption<br>• Air, water and solid waste pollution<br>• Generation of wastewater<br>• Noise and aesthetics pollution<br>• Material recovery<br>• Complying with environmental protection standards<br>• Tonnes CO_2 equivalent avoided<br>• Environment-related certificates<br>• Green process planning<br>• Use of harmful materials<br>• Lack of knowledge sharing<br>• Resource and environmental management capabilities<br>• Biodiversity conservation<br>• Collaboration with environmental partners<br>• Environmental management systems<br>• Green image<br>• Ecotechnological maturity	• Transportation cost<br>• Total logistical costs<br>• Cost of materials<br>• Investment cost<br>• Operational cost<br>• Collection cost<br>• Profit from reused products<br>• Land costs<br>• Convenience of the possessed technology<br>• Cost of sorting waste<br>• Cost of imports and exports<br>• Financial stability<br>• Innovation and generation<br>• of new economic activities<br>• Inspection/sorting and disassembly cost<br>• Cost of collection<br>• Cost of disposal<br>• Financial barriers<br>• Personnel costs<br>• Energy prices<br>• Purchasing price of WEEE<br>• Market exploration<br>• Competitiveness<br>• Market competition
**Social**	**Technical**
• Social acceptance<br>• Employment opportunities<br>• Hygiene & Safety<br>• Potential of new job creation<br>• Social inclusion<br>• Generation of income<br>• Access to health care<br>• Access to education<br>• Social responsibility<br>• Lack of public awareness<br>• Sociocultural barriers<br>• Social network<br>• Regulatory compliance	• State of the technology<br>• Recyclable<br>• Flexibility<br>• Design<br>• Process safety<br>• Lead time<br>• Reject rate<br>• Market credibility<br>• Performance history<br>• Strength<br>• Durability<br>• Existence of infrastructure<br>• Existence of other recycling plants

• Organizational culture<br>• Social conflict<br>• Corporate reputation	• Existence of WEEE<br>• Capacity need<br>• Technical capability<br>• Experience & qualification<br>• Problem-solving ability<br>• Quality of service<br>• Responsiveness and flexibility<br>• % recoverable material<br>• % recyclable material<br>• Residual/market value of components<br>• Working day utilization<br>• Vehicle utilization<br>• Total distance travelled<br>• Ease of disassembly<br>• WEEE storage capacity<br>• Capability of R&D
Legal	
• Legislative needs<br>• Extended producer responsibility (EPR) level<br>• Application of priorities of legislation<br>• Policy and regulatory barriers<br>• Regulatory obligations and risk compliances<br>• Waste disposal subsidy<br>• Support from local authorities<br>• Tariffs and tax preferences<br>• Environmental grants	

Table S2. Case study description.

	Location	Description
[67]	Greece	Development of two UTRs in Greece in addition to other existing WEEE treatment facilities
[7]	Greece	Designing the RL network, considering 37 major collection points and two treatment facilities in the cities of Thessaloniki and Polikastro
[42]	Canada	Designing and optimizing a closed-loop supply chain (CLSC) network for mobile phones, determining the location and number of facilities, the amount of required materials to produce a mobile phone and the number of products that should be transported between various facilities
[72]	Greece	Evaluation of nine alternative WEEE management scenarios
[43]	China	Mitigating the pollution caused by the release of hazardous materials in the informal WEEE recycling
[64]	Turkey	With the aim of satisfying national requests, according to researches of the Ministry of Environment and Urbanism in Turkey, 850 collection points for the district municipalities and 15 large collection centres are needed
[28]	Turkey	Application of the multi-criteria approach to a telecommunication enterprise that produces and sells cellphones
[73]	France	Application of the methodology to the case of printed wiring board (PWB) recycling in France
[61]	Turkey	Application of the method to a privately held chemical company located in Turkey
[36]	Specific case study	Analysis of a specific case study considering a typical treatment plant that manages all categories of WEEE
[37]	Brazil	Assessment of sustainability and prioritizing system alternatives for potential implementation in the metropolitan region of Rio de Janeiro
[62]	Italy	A WEEE transportation network in the north of Italy is addressed by means of an integrated solution approach
[14]	Europe	Application to the current European situation, in order to reduce import dependency
[70]	Specific case study	The developed methodology is demonstrated through its application in a real-world case study of an ISDN network terminal
[5]	Australia	Identification of potential candidate products that are outside of the actual scheme
[44]	India	A real-life case study of a mobile phone manufacturing company is presented to demonstrate the steps of the decision support system
[45]	Turkey	Application for selection of an outsourcing firm for the management of WEEE in Istanbul
[46]	Iran	Application for prioritizing electronic waste management options in Tehran
[47]	Korea	Assessing the potential of waste electronic devices or products that may

		become target WEEE for recycling and management in Korea
[41]	Poland	Assessment of WEEE take-back in a suburb of Tychy (Poland)
[48]	India	Identification of 10 barriers that hinder the successful implementation of e-waste management in the Indian context
[9]	India	Assessment of the barriers related to adoption of WEEE management from a multiple-stakeholder perspective
[49]	India	Assessment of an Indian electronics company (ABC) located in the western region of India
[75]	India	Assessment of the case study of Mumbai
[65]	Specific case study	Evaluation of a laboratory-based robotic disassembly cell and three automotive electronic components
[60]	Poland	Application through a case study in Opole (Poland)
[18]	India	Evaluation of an Indian electronics industry
[50]	India	Evaluation of an Indian electronics company
[63]	Spain	Assessment of potential new plants in south and central Spain
[68]	India	Evaluation of the possibility of using e-waste to develop a new specialized recycled product for the market
[74]	Cyprus	Comparison of 12 alternative management systems, ranked according to their performance and efficiency
[51]	Iran	Evaluation of the proposed model through a real case in Iran
[52]	India	Evaluation of the proposed model through a real case in the Mumbai area
[19]	Thailand	Application of the proposed method through the case of Thailand's electronics industry
[53]	UK	Assessment of manufacturers in the United Kingdom and determination of the waste volumes available within these companies
[54]	Specific case study	Application of a case regarding service outsourcing related to transportation
[6]	Ghana	Assessment of the approach to minimize risk and recover value from WEEE
[55]	Brazil	Assessment and classification of the internal and external barriers
[66]	Sri Lanka	Evaluation of the most suitable e-waste recycling programme
[56]	Specific case study	Evaluation of different innovation strategies for increasing competitiveness and achieving sustainable development
[57]	Specific case study	Assessment of the sustainable planning approach for e-waste recycling jobs in an e-recycling company
[69]	India	Electing a sustainable location of WEEE recycling plant
[58]	India	Modelling the e-waste mitigation strategies
[59]	Brazil	Reverse Logistic model
